# Early changes in somatosensory function in spinal pain: protocol for a systematic review

**DOI:** 10.1186/2046-4053-2-90

**Published:** 2013-10-02

**Authors:** Anna Marcuzzi, Catherine M Dean, Julia M Hush

**Affiliations:** 1 Discipline of Physiotherapy, Faculty of Human Sciences, 75 Talavera Rd, Macquarie University, North Ryde, New South Wales 2109, Australia

**Keywords:** Pain, Back pain, Neck pain, Acute pain, Subacute pain, Somatosensory function, Sensitization, Sensory testing

## Abstract

**Background:**

Back and neck pain are common conditions that have a high burden of disease. Changes in somatosensory function in the periphery, the spinal cord and the brain have been well documented at the time when these conditions have become chronic. It is unknown, however, how early these changes occur, what the timecourse is of sensory dysfunction and what the specific nature of these changes are in the first 12 weeks after onset of pain. In this paper, we describe the protocol for a systematic review of the literature on somatosensory dysfunction in the first 12 weeks after pain onset.

**Methods and design:**

We will conduct a comprehensive search for articles indexed in the databases Ovid MEDLINE, Ovid Embase, Ovid PsycINFO and Cochrane Central Register of Controlled Trial (CENTRAL) from their inception to August 2013 that report on any aspect of somatosensory function in acute or subacute neck or back pain. Two independent reviewers will screen studies for eligibility, assess risk of bias and extract relevant data. Results will be tabulated and a narrative synthesis of the results conducted.

**Discussion:**

Currently, there is a gap in our knowledge about the timing of somatosensory changes in back and neck pain. The systematic review outlined in this protocol aims to address this knowledge gap and inform developments in diagnostic tools and pain mechanism-based treatments.

**Trial Registration:**

Our protocol has been registered on PROSPERO, CRD42013005113.

## Background

Back and neck pain are acknowledged as common health problems affecting nearly everyone at some point in their life [1]. Although pain reduces rapidly in the first 1 to 2 months for some individuals after an acute onset, approximately two-thirds of people do not recover [2]. For those who develop disabling chronic pain, the associated personal and societal burden is high [3].

Current treatments for back and neck pain do not result in outcomes that are much better than the natural course of the condition [2]. Over the last few decades, there has been an emphasis on research to unravel the mechanisms that contribute to the pathogenesis of chronic pain to inform the development of more effective treatments [4,5]. There is now a great deal of evidence that multiple changes in the somatosensory nervous system characterize chronic spinal pain (for a review, see [6]). For example, recent studies have shown that positive and negative sensory phenomena such as increased pain sensitivity [7-9], allodynia or hypoesthesia [10] as well as alterations in body perception [11,12] are commonly encountered in chronic back and neck pain patients, reflecting functional and structural changes at different levels along the neuraxis. In addition, functional imaging studies provide evidence of central pain amplification and cortical reorganization in low back pain, which correlate with clinical manifestations observed in these patients [13,14]. These changes in somatosensory function impact on, and are affected by, cognitive and behavioral factors such as catastrophizing, fear, and anxiety, which dynamically interact to modulate and facilitate the experience of pain.

It is therefore well documented that somatosensory dysfunction characterizes chronic spinal pain conditions; however, it is not fully elucidated how early these changes occur in back and neck pain. The review outlined in this protocol will explore changes in somatosensory function in acute and subacute spinal pain populations in order to address this gap in current knowledge.

### Research questions

This literature review aims to answer the following research questions: (1) Have changes in somatosensory function been detected in the first 12 weeks of spinal pain? (2) How early has somatosensory dysfunction been detected in spinal pain? And (3) What type of somatosensory changes have been detected in spinal pain?

## Methods and design

### Study registration

The protocol of this systematic review has been registered on PROSPERO 2013 [15] (registration number: CRD42013005113).

The systematic review protocol has been conducted and reported using the Preferred Reporting Items for Systematic Reviews and Meta-Analyses (PRISMA) statement guidelines [16].

### Search strategy for identification of relevant studies

To identify the relevant literature, electronic searches will be conducted in the following databases: Ovid MEDLINE, Ovid Embase, Ovid PsycINFO and Cochrane Central Register of Controlled Trial (CENTRAL) from their inception to August 2013. A comprehensive search strategy has been designed with the assistance of an experienced research librarian and adjusted to account for differences in indexing across databases. The updated search strategy of the Cochrane Back Review Group 2013 [17] was used to identify spinal pain terms, which were combined with relevant keywords for the somatosensory function domain (Appendix 1). Articles identified through reference lists of included studies and relevant systematic reviews will be considered for inclusion based on their title. Non-English language studies will be included, where a translation can be made available.

### Eligibility criteria

#### Participants

We will include studies of adults (18 years or older) with acute or subacute (up to and including 12 weeks) spinal pain (back or neck pain). Studies will be excluded if the participants have spinal pain due to serious pathologies (for example, fracture, neoplasm, infection, failed back surgery syndrome) or specific conditions (for example, rheumatoid arthritis, fibromyalgia, spondylolisthesis, pregnancy and postpartum) or who have had spinal surgery. Studies will also not be included if they report on a mixed population of chronic and acute or subacute spinal pain where the results for acute or subacute participants cannot be extracted separately.

#### Outcome measures

The outcomes of interest are any measure of somatosensory dysfunction (for example, hyperalgesia, allodynia, dysaesthesia, neuropathic pain) assessed by any experimental or clinical examination, by quantitative sensory testing or by any relevant questionnaire, reported within the first 12 weeks of onset of back or neck pain.

#### Types of study

We will include relevant study designs such as cross-sectional studies, surveys, case-control studies, randomized controlled trials and observational studies. Qualitative studies and retrospective studies will be excluded. We will exclude intervention studies if assessment of somatosensory function is only reported after treatment (for example, drug administration, surgical techniques). Reference lists of relevant systematic reviews will be checked in order to identify relevant primary studies, but systematic reviews will otherwise be excluded.

### Screening of studies

After removal of duplicate papers, identification of studies that meet the inclusion criteria will be independently conducted by two reviewers based on the title and then abstract. Reasons for exclusion of papers will be recorded when screening full papers. Papers of the resulting studies will be reviewed independently by two reviewers for their eligibility using a standardized eligibility sheet. Any disagreement arising between the reviewers will be resolved by discussion and consensus and with the assistance of a third reviewer at all stages of screening.

### Data extraction

Data from included studies will be extracted independently by two reviewers using a standardized data extraction form. Differences in data extraction will be resolved by consensus and the assistance of a third reviewer. Authors of studies will be contacted if data are incomplete or clarification is required. The following data will be extracted from each included study. General study information: authors, year of publication, language; study design: cross-sectional, survey, case-control, observational study or clinical trial; clinical setting: primary care, specialist clinic, hospital outpatient department; population characteristics: demographic information (age, gender); case definition and description: classification or diagnostic criteria used, region of pain (lumbar, cervical, mixed), duration of pain, severity of pain, functional status, comorbidities, medications; somatosensory function: data from psychophysical measures, clinical assessment or description, questionnaire at specified time points from onset of spinal pain for spinal pain and control cohorts (where described).

### Risk of bias assessment

We were unable to identify an existing instrument suitable to assess the risk of bias for the different study types eligible for this review. Therefore, study quality will be assessed using a system adapted from Lewis *et al*. [18] and Tesarz *et al*. [19], designed to evaluate study features most relevant to the current review. These features are: (1) that the sample was clearly described; (2) that the sample was representative of the target population; (3) that the somatosensory assessment method used was standardized, validated and fully described; (4) that there was blinding of those assessing somatosensory function to group allocation (where relevant); and (5) that factors known to influence pain assessment were evaluated or controlled for in the analysis (for psychophysical studies). For this last item, known confounders include medication use, caffeine intake prior to testing, comorbid pain condition, different testing times during the day and phase of menstrual cycle (females) [18]. Each risk of bias item will be evaluated as outlined in Table 1, by two reviewers and any disagreement discussed with a third reviewer to reach consensus. Studies will be considered to have high risk of bias if the majority of relevant criteria are not satisfied.

**Table 1:** Risk of bias assessment

**Category**	**Criteria**	**Judgment**
Defined sample	Inclusion/exclusion criteria were clearly specified	Yes
	Comment:	No
		Unsure
		N/A
Representative sample	Clinical and demographic characteristics were well described	Yes
	Comment:	No
		Unsure
		N/A
	Recruitment procedure was specified (including source population) and appropriate	Yes
	Comment:	No
		Unsure
		N/A
Somatosensory assessment	Somatosensory assessment method was standardized or validated	Yes
	Comment:	No
		Unsure
		N/A
	Method of somatosensory assessment was fully described	Yes
	Comment:	No
		Unsure
		N/A
Blinding of assessment	Assessment of somatosensory function was blinded to participant group or condition	Yes
	Comment:	No
		Unsure
		N/A
Controlled risk of known confounders	Factors known to influence pain assessment were evaluated or controlled for	Yes
	Comment:	No
		Unsure
		N/A

### Data analysis

It is anticipated that the studies will be too heterogeneous in multiple domains to allow any data pooling or quantitative synthesis. Data will be therefore gathered and presented in a table and a narrative synthesis of the findings will be conducted. Where possible, an indication of the timeline of changes in somatosensory function will be presented. Because of the anticipated heterogeneity of studies, it is unlikely that quantitative analyses based on study quality will be possible. Therefore, the risk of bias assessment of included studies will be summarized in a table and results and implications will be critically discussed.

## Discussion

This systematic review will fill an important gap in our current knowledge about the timecourse and nature of changes in somatosensory function that occur in the early stages of back and neck pain, which may be instrumental in the development of disabling chronic pain. An improved understanding of the timing and onset of sensory dysfunction will enable clinicians and researchers to develop more effective diagnostic tools and mechanism-based treatments to prevent the development of chronic back and neck pain.

## Appendix 1: Ovid MEDLINE search strategy

1. back pain/

2. low back pain/

3. back disorder*.mp.

4. (lumbar adj pain).ti,ab.

5. sciatica/

6. sciatic neuropathy/

7. Intervertebral Disc Degeneration/

8. (disc adj prolapse).ti,ab.

9. (disc adj herniation).ti,ab.

10. (facet adj joint*).ti,ab.

11. backache.ti,ab.

12. dorsalgia.mp.

13. or/1-12

14. Neck Pain/

15. whiplash injur*.mp.

16. exp Neck Injuries/

17. Neck Muscles/

18. neck.ti,ab.

19. or/14-18

20. (femur or humerus).mp. [mp = title, abstract, original title, name of substance word, subject heading word, keyword heading word, protocol supplementary concept, rare disease supplementary concept, unique identifier]

21. 19 not 20

22. exp Pain Perception/

23. pain, referred/

24. sensory profile*.mp. [mp = title, abstract, original title, name of substance word, subject heading word, keyword heading word, protocol supplementary concept, rare disease supplementary concept, unique identifier]

25. Analgesia.ti,ab.

26. allodynia.ti,ab.

27. neuralgia/

28. sensory hypersensitivity.ti,ab.

29. hyperpathia.ti,ab.

30. exp somatosensory disorders/

31. hyp?algesia.ti,ab.

32. peripheral sensit*.ti,ab.

33. central pain.ti,ab.

34. quantitative sensory test*.mp.

35. experim* pain.mp.

36. (pain adj test*).mp.

37. bedside exam*.mp.

38. psychophysic*.mp.

39. (neuropathic pain questionnaire or painDETECT or DN4 or NPSI or PQAS or ID-pain or LANSS).ti,ab. [mp = title, abstract, original title, name of substance word, subject heading word, keyword heading word, protocol supplementary concept, rare disease supplementary concept, unique identifier]

40. temporal summation.ti,ab.

41. wind up.ti,ab.

42. two-point discrimination.ti,ab.

43. (second adj pain).ti,ab.

44. tactile acuity.ti,ab.

45. diffuse noxious inhibitory control.mp.

46. conditioned pain modulation.mp.

47. pain threshold/

48. central sensit*.ti,ab.

49. Nociceptors/

50. ((pressure or thermal or cold or heat or eletrical or mechanical) adj pain).ti,ab. [mp = title, abstract, original title, name of substance word, subject heading word, keyword heading word, protocol supplementary concept, rare disease supplementary concept, unique identifier]

51. ((cold or warm) adj detection).ti,ab.

52. (pain adj tolerance).ti,ab.

53. (detection adj threshold).ti,ab.

54. 13 or 21

55. or/22-53

56. 54 and 55

57. 56 not surg*.mp.

58. qualitative research/

59. retrospective studies/

60. 58 or 59

61. 57 not 60

62. limit 61 to humans

## Abbreviations

CENTRAL: Cochrane Central Register of Controlled Trials; PROSPERO: Prospective Registering of Systematic Reviews; PRISMA: Preferred Reporting Items for Systematic Reviews and Meta-analyses.

## Competing interests

The authors declare they have no competing interests.

## Authors’ contributions

AM is the lead researcher of this project, supported by doctoral supervisors JMH and CMD. AM, JMH and CMD all contributed to the development of the protocol. AM and JMH led the writing of the protocol manuscript. All authors critically revised the protocol and read and approved the final version.
